# Lattice Boltzmann Simulation of the Hydrodynamic Entrance Region of Rectangular Microchannels in the Slip Regime

**DOI:** 10.3390/mi9020087

**Published:** 2018-02-16

**Authors:** Niya Ma, Zhipeng Duan, Hao Ma, Liangbin Su, Peng Liang, Xiaoru Ning, Boshu He, Xin Zhang

**Affiliations:** 1 School of Mechanical, Electronic and Control Engineering, Beijing Jiaotong University, Beijing 100044, China; 15121339@bjtu.edu.cn (N.M.); 16121364@bjtu.edu.cn (H.M.); 16116368@bjtu.edu.cn (L.S.); 14121328@bjtu.edu.cn (P.L.); 17121369@bjtu.edu.cn (X.N.); hebs@bjtu.edu.cn (B.H.); zhangxin@bjtu.edu.cn (X.Z.); 2 Beijing Key Laboratory of Powertrain for New Energy Vehicle, Beijing Jiaotong University, Beijing 100044, China

**Keywords:** lattice Boltzmann equation method (LBE), slip flow, entrance region, rectangular microchannels, apparent friction factor and Reynolds number product, hydrodynamic development length

## Abstract

Developing a three-dimensional laminar flow in the entrance region of rectangular microchannels has been investigated in this paper. When the hydrodynamic development length is the same magnitude as the microchannel length, entrance effects have to be taken into account, especially in relatively short ducts. Simultaneously, there are a variety of non-continuum or rarefaction effects, such as velocity slip and temperature jump. The available data in the literature appearing on this issue is quite limited, the available study is the semi-theoretical approximate model to predict pressure drop of developing slip flow in rectangular microchannels with different aspect ratios. In this paper, we apply the lattice Boltzmann equation method (LBE) to investigate the developing slip flow through a rectangular microchannel. The effects of the Reynolds number (1 < *Re* < 1000), channel aspect ratio (0 < *ε* < 1), and Knudsen number (0.001 < *Kn <* 0.1) on the dimensionless hydrodynamic entrance length, and the apparent friction factor, and Reynolds number product, are examined in detail. The numerical solution of LBM can recover excellent agreement with the available data in the literature, which proves its accuracy in capturing fundamental fluid characteristics in the slip-flow regime.

## 1. Introduction

In recent years, rapid development in manufacturing technologies, together with a motivation toward MEMS [1,2,3,4], have attracted much academic research of laminar flows in relatively short channels with different cross-sections. It is of paramount importance that the velocity profiles undergo rapid transformations from essentially uniform inlet profiles to fully-developed parabolic profiles. The small length scales commonly encountered in microfluidic devices suggest that rarefaction effects are significant, where the hydrodynamic development length may be in the same magnitude of the microchannel length, entrance effects have to be taken into account.

Rarefaction effects, usually characterized by the Knudsen number, which is defined as the ratio of the molecular mean free path of gas to a characteristic dimension of flow domain. According to the value of Kn, the flow can be classified into four flow regimes, the continuum flow (Kn ≤ 0.001), the slip flow (0.001 < Kn ≤ 0.1), the transition flow (0.1 < Kn ≤ 10), and free molecular flow (Kn *>* 10). Here we pay special attention on the slip flow, where the rarefaction effect may be significant, and the conventional Navier–Stokes simulation can be used with appropriate s velocity slip boundary conditions. This regime has attracted extensive attention from the engineering research community because of its significance associated with many of the practical microfluidic devices. It has been the subject of many studies employing continuum modeling to examine the slip-flow convection at the microscale [5,6,7,8] over the past two decades.

Liquid slip is very important in microfluidic devices with a superhydrophobic surface because it reduces the required pressure in pressure-driven flows. Due to the lack of molecular based theory of liquids, a nondimensional number (modified Knudsen number $Kn^{*}$)is not commonly used for liquids. When considering liquids, the molecular mean free path may be replaced by the slip length or the molecular interaction length. To look at it from a slightly more general mathematical viewpoint, when no slip condition on the solid surfaces is partially relaxed, the molecular mean free path and the term involving the accommodation coefficient (λ(2−σ)/σ) and the slip length ($\lambda_{s}$) for liquids have the identical mathematical meaning. This could be significant because of a shortage of information for apparent liquid slip flow in the literature. The results in this paper can also be applied in the research of the liquid slip flow.

## 2. Literature Review

There is extensive literature appearing on micro-scale slip flow. Most of the numerical, experimental and analytical studies were performed using the Navier–Stokes and energy equations with modified boundary conditions that consider the rarefaction effects on the velocity and temperature fields. Arkilic et al. [7,9] performed analytical and experimental studies in gaseous flow through microchannels. In their work, they obtained analytical solution of Navier–Stokes equations at low Kn with a first-order slip flow boundary condition for two dimensional microchannel flow and presented the general pressure and velocity profiles. Experiments conducted by Arkilic et al. [7,9], revealed that the good agreement between the analytical solutions obtained from the Navier–Stokes equations and experimental data, and confirmed that classical Navier–Stokes and energy equations can still be used in microchannel flows, with modified boundary conditions.

Chen et al. [10] numerically examined the two-dimensional, compressible Navier–Stokes equations with a first-order velocity slip boundary condition for gaseous flow in a microchannel. They concluded that the deviation between the measured friction factor and the conventional theory was caused by the rarefaction effects.

Morini et al. [11] studied slip flow in rectangular microchannels. They presented the two dimensional velocity distribution of steady, laminar slip flow, for Newtonian fluids in a hydrodynamically fully-developed region [11]. The rarefaction effects on the pressure drop for an incompressible flow through silicon microchannels with rectangular, trapezoidal and double-trapezoidal cross-sections have been investigated [12]. They also pointed out the influence of the Knudsen number and the cross-section aspect ratio on the friction factor (fRe) reduction due to the rarefaction [13].

Vocale and Spiga [13] investigated rarefaction effects in the hydrodynamic entrance region of rectangular microchannels, for slip gaseous flows and calculated the main physical parameters. In their work, apparent Poiseuille number $Po_{app}$, incremental pressure drop number or Hagenbach’s factor K(∞), pressure and velocity profiles were also presented.

Renksizbulut et al. [14] had numerically studied rarefied gas flow in the entrance region of rectangular microchannels in the slip-flow regime. They examined the effects of Reynolds number, channel aspect ratio, and Knudsen number on the developing velocity fields, the entrance length, and the friction coefficient in detail.

The flow characteristic of water in microchannels with rectangular cross-sections has been studied by Jiang et al. [15]. They found that the experimental data of the friction factor were larger than the values of the conventional theory. They explained that this phenomenon was conducted by the significant hydrodynamic entrance region effects in short microchannels.

Steinke and Kandlikar [16] reported a database evaluating experimental data related to friction factors in microchannels, and carried out comparisons and analyses on the fundamental research topic of fluid flow and heat transfer in microchannels. They have presented an explanation for the deviation between the experimental data and the predicted theoretical values that the past papers do not account for the entrance and exit losses and the developing flow in the microchannel.

Li et al. [17] investigated the entrance effects on flow characteristics in micro-scale channels experimentally and numerically. They found that when the channel got shorter, the friction constant became higher and attributed the entrance effects as one of the critical factors leading to this phenomenon.

Palm [18] published a review regarding heat transfer and friction factor in single phase flow in microchannels. The experimental results obtained in microchannels deviated from conventional macrochannels. Hence, they pointed out that entrance effects cannot be neglected in some cases where fluid flow in channel might be not fully developed.

Mishan et al. [19] compared their experimental data for developing flow in rectangular microchannels with theoretical solutions and numerical data from literature. They confirmed that the results of pressure drop and heat transfer accounting for the entrance effects were consistent with the conventional theoretical solutions for water in microchannels, however the results differed from other researchers. They emphasized on common phenomena for standard flows, such as the profile of inlet velocity, axial heat conduction, effect of the design inlet and outlet manifolds, which were often ignored but not the concern of entrance effects by other researchers.

Zhang et al. [20] analyzed the effects of the hydrodynamically developing flow on the performance of the whole microchannel flow by means of the conventional equivalent method, the subsectional integral method, and experimental method. They made comparisons on the calculated results obtained from these three methods. They pointed out that the influence of the hydrodynamically-developing flow should be taken into consideration, and the apparent friction factor accounting for the pressure drop due to the friction was employed to replace the Fanning friction factor.

Chai et al. [21] performed experiments to measure the apparent friction factor and Reynolds number product $f_{app}Re$ at an aspect ratio of 0.5 for the entrance region in a rectangular microchannel under no-slip condition.

Recently, an alternative method the lattice Boltzmann method (LBM) has attracted a variety of researchers to apply it to simulate microchannel flows [22,23,24,25,26]. Advances in micro-electrical mechanical system (MEMS) and nanotechnology have spurred interest in utilizing LBM for the simulation of microfluidics.

Chen and Doolen [25] first applied the lattice Boltzmann method to micro-scale flow. In 2002, Nie et al. [26] first simulated a rarefied gas flow in micro-channel using the lattice Boltzmann method. Comparing simulation results with experiments, they discovered the slip velocity, the nonlinear pressure drop in micro-channel had a quadratic flow dependence on Knudsen number. In the same year, Lim et al. [27] investigated two-dimensional isothermal microchannel flow driven by a pressure difference by means of LBM. They used the specular bounce-back rule and the extrapolation scheme to simulate slip effects, and their results of 2D channel flows compared well with Arkilic’s experimental data and analytical solutions [27].

Dorari et al. [28] investigated the characteristics of the flow and heat transfer in microchannels using the lattice Boltzmann method, in which the effects of rarefaction and wall roughness on gaseous flow and heat transfer were studied.

Succi [29] proposed a combination of the bounce-back and specular-reflection conditions to investigate the effects of slip motion on the solid wall. The results illustrated that the value of the bounce-back coefficient may significantly affect the slip flow velocity.

Sbragaglia and Succi [30] simulated the rarefaction and compressibility effects of gas microchannel flows by defining specular factor, *r* = 0.59, in the specular bounce-back scheme. They exhibited a mathematical formulation of kinetic boundary conditions for LBE schemes, in terms of reflection, slip, and accommodation coefficients.

With an equilibrium wall boundary condition, Lee and Lin [31] employed an implicit lattice Boltzmann equation (LBE) method and derived a *Kn*-*τ* relation that differs from that of Lim et al. [27] by a factor of 0.5. They simulated the rarefaction and compressibility effects of gas microchannel flows, demonstrated the computed slip velocity was in excellent agreement with the analytical formula by Arkilic et al. [7].

Jeong et al. [32] applied the lattice Boltzmann equation method to study the compressibility and rarefaction of three-dimensional (3D) slip flow in rectangular microchannels. The influence of channel aspect ratio and outlet Knudsen number on pressure nonlinearity, slip velocity, and mass flow rate were investigated. Their results agree well with parallel plate microchannel limiting cases.

Tang et al. [33] later simulated the micro-Couette flow, the micro-Poiseuille flow with the combination of the kinetic boundary condition, and the specular reflection boundary condition. The analysis studied by Guo et al. [34] indicated the wall effect on the relaxation time should be considered in the LBE for modeling micro-gas flows, and they further summarized systemically the applicability of the present LBGK models for gas flows in microchannels.

As shown by Guo and Zheng [35], Verhaeghe et al. [36], the lattice Boltzmann method can give a fairly good description of gas flows in the slip regime for microflows. Zhang et al. [37] implemented the tangential momentum accommodation coefficient to describe the gas-wall interaction in a LBE (D2Q9) model.

Montessori et al. [38,39,40,41] had done a lot of valuable work on high-Knudsen flows. The thorough research focused on the non-equilibrium hydrodynamic effects of complex nanoscale flow of two dimension and three dimension in porous media across a wide range of Knudsen numbers beyond the general hydrodynamic limits, employing lattice Boltzmann realization of Grad’s extended hydrodynamic approach and lattice Boltzmann method (LBM) with thermodynamic models. Falcucci et al. [42,43] first employed the LB method for pulsed, chemically-active flows with a new boundary condition to account for catalytic reactions at the gas–solid interface in the transitional slip-flow regime. The results indicated that the LBM can provide computational guidance to the design of catalytic devices operating the pulsed conditions at high Knudsen numbers (0.1 < Kn < 1). The complex hydrodynamic problems across a broad range Knudsen number were studied intensively. The aforementioned articles [38,39,40,41,42,43] have important reference and guidance value to the current work and future studies.

Owing to its kinetic characteristics and distinctive advantages over conventional numerical methods, the LBM has been a new potential tool and attained great success in studying microflows. To the authors’ best knowledge, there are no systematical investigations reported in using LBM to study the developing laminar flow for rectangular microchannels in the slip regime, especially focusing on the aspect ratios and hydrodynamic development length of rectangular microchannels.

In the open literature, the only fluid flow data available for the laminar entrance region of rectangular microchannels may be the model proposed by Duan and Muzychka [44,45], which is in terms of the geometry of the cross-section, the Knudsen number, and the accommodation coefficient for predicting the friction factor and Reynolds number product, and has demonstrated an approximate accuracy of 10% for the most common duct shapes in microchannel applications.

The aim of this paper is to analyze hydrodynamic characteristics of the entrance region for laminar flow in three-dimensional rectangular ducts with aspect ratios from 0.1 to 1. By means of LBE method, we concentrate on the effects of rarefaction on the hydrodynamically-developing flow and pressure drop in the entrance region for rectangular ducts. In the present investigation, the role of the Reynolds number, aspect ratios and Knudsen number on the hydrodynamic development length of rectangular microchannels is also examined and investigated.

## 3. Models

### 3.1. Mathmatical Models of Slip Flow through Rectangular Microchannels

It is assumed that this internal flow is three-dimensional, incompressible, laminar, isothermal, and steady in the entrance region of rectangular microchannels. There exists a velocity slip on the wall. Assuming the working fluid is a Newtonian fluid, according to the proposed hypotheses, the continuity equation and momentum equation in its most common general form are:

(1) $$\frac{\partial \rho }{\partial t}+\rho (\frac{\partial u}{\partial x}+\frac{\partial v}{\partial y}+\frac{\partial w}{\partial z})=0$$

(2) $$\frac{\partial \rho u}{\partial t}+\rho (u\frac{\partial u}{\partial x}+v\frac{\partial u}{\partial y}+w\frac{\partial u}{\partial z})=-\frac{\partial p}{\partial x}+\mu (\frac{\partial^{2}u}{\partial x^{2}}+\frac{\partial^{2}u}{\partial y^{2}}+\frac{\partial^{2}u}{\partial z^{2}})$$

(3) $$\frac{\partial \rho v}{\partial t}+\rho (u\frac{\partial v}{\partial x}+v\frac{\partial v}{\partial y}+w\frac{\partial v}{\partial z})=-\frac{\partial p}{\partial y}+\mu (\frac{\partial^{2}v}{\partial x^{2}}+\frac{\partial^{2}v}{\partial y^{2}}+\frac{\partial^{2}v}{\partial z^{2}})$$

(4) $$\frac{\partial \rho w}{\partial t}+\rho (u\frac{\partial w}{\partial x}+v\frac{\partial w}{\partial y}+w\frac{\partial w}{\partial z})=-\frac{\partial p}{\partial z}+\mu (\frac{\partial^{2}w}{\partial x^{2}}+\frac{\partial^{2}w}{\partial y^{2}}+\frac{\partial^{2}w}{\partial z^{2}})$$

The velocity distribution must be satisfactory for the slip boundary condition at the walls. According to gas kinetic theory, the gas velocity at the wall is different from the wall velocity, which is expressed in terms of the local velocity gradient at the wall. The first-order Maxwell boundary condition for the velocity at the wall is:

(5) $$u=-\lambda \frac{2-\sigma }{\sigma }{\left(\frac{\partial u}{\partial n}\right)}_{wall}$$

where *λ* is the molecular mean free path, σ represents the tangential momentum accommodation coefficient, which can vary between zero and unity. Here, the subscript wall identifies a wall surface with normal coordinate *n*.

Since the pressure gradients found in microchannels are quite high, the flow lengths are generally kept low. To account for the developing region, the pressure drop equations are presented in terms of an apparent friction factor $f_{app}$, which represents a true value of the friction factor from the entrance region to the local position along the flow direction under calculation. Thus, the pressure drop involved the apparent friction factor $f_{app}$ can be written as:

(6) $$\Delta p=\frac{2f_{app}\rho u_{m}^{2}x}{D_{h}}$$

(7) $$\frac{\Delta p}{\rho u^{2}/2}=\frac{4x}{D_{h}}f_{app}=4x^{+}f_{app}Re$$

For the slip flow entrance region problem, the available data are given by the approximately analytical solutions presented by Duan and Muzychka [45], where they presented a comprehensive review of laminar flow in ducts of various cross-sections, including rectangular channels. We can obtain the apparent friction factor and Reynolds number product from Duan and Muzychka [45]:

(8) $$f_{app}Re=\text{​}\frac{24}{{\left(1+\varepsilon \right)}^{2}\{1-\frac{192\varepsilon }{\pi^{5}}\left[\tanh(\frac{\pi }{2\varepsilon })+\frac{1}{243}\tanh(\frac{3\pi }{2\varepsilon })\right]\}}+\frac{1}{3x^{+}}-2\sum_{i=1}^{\infty }\frac{\left[3-\exp(4\alpha_{i}^{2}x^{+})]\exp(4\alpha_{i}^{2}x^{+}\right)}{\alpha_{i}^{2}x^{+}}$$

definition of a non-dimension flow distance is:

(9) $$x^{+}=\frac{x}{D_{h}Re}$$

Here, $x^{+}$ is the dimensionless duct length, and x is the local position in the flow direction. The Reynolds number is defined as $Re=\rho u_{m}D_{h}/\mu$, μ is the fluid viscosity, $D_{h}$ is the hydraulic diameter of a rectangular channel defined as four times the cross-sectional flow area over the wetted perimeter $D_{h}=4A/P=2ab/(a+b)$, ρ is the fluid density, and $u_{m}$ is the mean longitudinal velocity. In addition, ε is defined as the aspect ratio of rectangular cross section (ratio of the short side to the long side), and $\alpha_{i}$ are the eigenvalues which are based on the slip velocity boundary condition.

(10) $$f_{app}Re_{D_{h}}=\text{​}\frac{24}{{\left(1+\varepsilon \right)}^{2}\{1-\frac{192\varepsilon }{\pi^{5}}\left[\tanh(\frac{\pi }{2\varepsilon })+\frac{1}{243}\tanh(\frac{3\pi }{2\varepsilon })\right]\}}(\frac{1}{1+(11.97-10.59\varepsilon +8.49\varepsilon^{2}-2.11\varepsilon^{3})Kn})$$

For the semi-theoretical analytical model, we can use Equation (10) to investigate the apparent friction factor and Reynolds number product for developing slip flow in rectangular ducts. With arbitrary aspect ratios and axial distances, it is of great convenience to calculate the apparent friction factor and Reynolds number product $f_{app}Re$. The calculated results are used as a benchmark in the present study.

### 3.2. Lattice Boltzmann Model

#### 3.2.1. Lattice Boltzmann Equation and the Corresponding Macroscopic Equation

**Figure 1:**
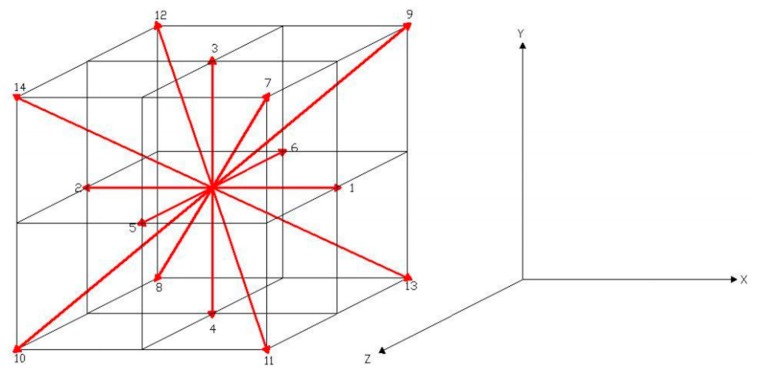
Schematic illustration of space discrete and velocity discrete in the D3Q15 model.

To demonstrate the utility of LBE method for simulating the three-dimensional flow in a rectangular channel, we consider the isothermal D3Q15 BGK-LBE (three-dimensional fifteen-velocity) model [23] to derivate the incompressible Navier–Stokes equation. The space discrete and velocity discrete of the D3Q15 model are shown in Figure 1.

For the square lattice LBGK model D3Q15, the basic discrete-velocity Boltzmann kinetic equation is:

(11) $$f_{i}(x+c_{i}\Delta t,t+\Delta t)=f_{i}(x,t)-\frac{1}{\tau }[f_{i}(x,t)-f_{i}^{eq}(x,t)],i=0,1,\ldots ,14$$

where $f_{i}^{eq}$ is the local-equilibrium distribution function and τ is the dimensionless collision relaxation time which fixes the rate of approach to equilibrium, Δx and Δt are the spatial separation of the lattice and time step size. In our axisymmetric D3Q15 model, the fifteen discrete velocities of our model are defined as follows:

(12) $$c_{i}=\left[\begin{array}{ccccccccccccccc} 0 & +1 & -1 & 0 & 0 & 0 & 0 & +1 & -1 & +1 & -1 & +1 & -1 & +1 & -1\\ 0 & 0 & 0 & 1 & -1 & 0 & 0 & +1 & -1 & +1 & -1 & -1 & +1 & -1 & +1\\ 0 & 0 & 0 & 0 & 0 & +1 & -1 & +1 & -1 & -1 & +1 & +1 & -1 & -1 & +1 \end{array}\right]$$

Here $c_{i}$ is the lattice molecular speed. In this model, we have assumed that $f_{i}(x,t)$ is the distribution function for particles with velocity $c_{i}$ at position x and time t. Macroscopic hydrodynamic variables such as mass density ρ, momentum density ρu, and pressure p can be expressed as:

(13) $$\sum_{i=0}^{14}f_{i}=\rho$$

(14) $$\sum_{i=0}^{14}f_{i}c_{i}=\rho u$$

(15) $$u=\frac{1}{\rho }\sum_{i=0}^{14}f_{i}c_{i}$$

(16) $$p=\frac{c^{2}}{3}\rho$$

where c is the particle streaming speed, $c=\sqrt{3RT_{0}}$, R is the specific gas constant, $T_{0}$ is the reference gas temperature, and c is assumed to have a value of unity.

The equilibrium distribution $f_{i}^{eq}$ of D3Q15 model is defined by Equation (17):

(17) $$f_{i}^{eq}(x,t)=w_{i}\rho \left[1-\frac{u^{2}}{2c_{s}^{2}}\right],\;i=0$$

(18) $$f_{i}^{eq}(x,t)=w_{i}\rho \left[1+\frac{c_{i}u}{c_{s}^{2}}+\frac{{\left(c_{i}u\right)}^{2}}{2c_{s}^{4}}-\frac{u^{2}}{2c_{s}^{2}}\right]\;i=1,2,\ldots ,14$$

where $\omega_{0}$ = 2/9 (*i* = 0), $\omega_{i}$ = 1/9 (*i* = 1, 2, 3, 4, 5, 6), $\omega_{i}$ = 1/72 (*i* = 7, 8, 9, 10, 11, 12, 13, 14), $c_{s}$ is speed of sound, usually taken as $c/\sqrt{3}$.

By undertaking a multiscale technique, we can recover the macroscopic equations of the model. As a result, it is considered as a numerical solver of the Navier–Stokes equation.

They are given by the continuity equation and the momentum equation:

(19) $$\frac{\partial \rho }{\partial t}+\nabla \cdot (\rho u)=0$$

(20) $$\frac{\partial \rho u}{\partial t}+\nabla \cdot (\rho uu)=-\nabla p+\rho \nu \nabla \cdot (\nabla u+{\left(\nabla u\right)}^{T})$$

In the LBM simulation, we must give appropriately the relationship between the relaxation time τ and the Knudsen number Kn to ensure the comparability of the results obtained experimentally, theoretically, and numerically [46].

The mean free-path λ can be in terms of the dynamic viscosity μ=ρv as:

(21) $$\lambda =\frac{\mu }{\rho }\sqrt{\frac{\pi RT}{2}}$$

Therefore, with Equation (21) we have:

(22) $$\lambda =(\tau -\frac{1}{2})\Delta t\sqrt{\frac{\pi RT}{2}}=\sqrt{\frac{\pi }{6}}(\tau -\frac{1}{2})\Delta x$$

from which we can obtain the relation between τ and Kn:

(23) $$\tau -\frac{1}{2}=\sqrt{\frac{\pi }{6}}NKn$$

In the LBGK model, N=h/Δx is the grid number in the characteristic length. The relaxation time *τ* and the kinematic viscosity of the fluid satisfy the relation $\upsilon =c_{s}^{2}(\tau -1/2)$. Note that, in our simulation, we generally adopt τ−1/2<<1, for the hydrodynamic region.

As usual, the LBE method consists of two steps, collisions and streaming. After one time step Δt, $f_{i}(x,t)$ will arrive at its neighboring lattice site $x+c_{i}\Delta t$ along the lattice velocity $c_{i}$. This process is usually called the streaming process. The streaming step is:

(24) $$f_{i}(x+c_{i}\Delta t,t+\Delta t)=f_{i}(x,t+\Delta t)$$

The collision step without forcing function is:

(25) $$f_{i}(x,t+\Delta t)=f_{i}(x,t)(1-\omega )+\omega f_{i}^{eq}(x,t)$$

For a certain site, there are other particles coming from different directions, collision occurs among them at this site, and the original particle numbers moving in each direction will be changed. In a LBM simulation, such streaming and collision processes are repeated again and again until satisfied results are achieved.

#### 3.2.2. Kinetic Boundary Conditions for LBE

Correct boundary conditions are crucially important for the accuracy and stability of the LBM. In this work, assuming a uniform velocity at the inlet of the microchannel, and the pressure is known and equal to 1 atm at the outlet boundary. Two types of boundary conditions must be dealt with: (a) the slip boundary conditions at the walls, (b) velocity boundary conditions at the inlet and pressure boundary conditions at the outlet. In the LBE techniques this flow can be simulated with the bounce-back of the non-equilibrium approach originally proposed by Zou and He [47] at the inlet. The velocity boundary condition for the model D3Q15 is applied as follows:

(26) $$f_{1}+f_{7}+f_{9}+f_{11}+f_{13}=\rho -(f_{0}+f_{2}+f_{3}+f_{4}+f_{5}+f_{6}+f_{8}+f_{10}+f_{12}+f_{14})$$

(27) $$f_{1}+f_{7}+f_{9}+f_{11}+f_{13}=\rho u_{x}+(f_{2}+f_{8}+f_{10}+f_{12}+f_{14})$$

(28) $$f_{1}=f_{2}+\frac{2}{3}\rho u_{x}$$

(29) $$u_{x}=1-\frac{1}{\rho }[f_{0}+f_{3}+f_{4}+f_{5}+f_{6}+2(f_{2}+f_{8}+f_{10}+f_{12}+f_{14})]$$

Non-equilibrium extrapolation scheme proposed by Guo et al. [48] is used here to deal with outlet pressure boundary conditions. According to this method, after the streaming step, the unknown density distribution functions at the node of the boundary are comprised of an equilibrium part and a non-equilibrium part:

(30) $$f_{i}(x_{w},t)=f_{i}^{eq}(x_{w},t)+[f_{i}(x_{f},t)-f_{i}^{eq}(x_{f},t)]$$

where $x_{w}$ is the nearest neighbor fluid node of boundary node $x_{f}$ ($x_{f}=x_{w}+c_{i}\Delta t$).

When the flow encounters the slip-flow regime, the velocity slip occurs. The velocity slip near the wall plays an important role, which makes the implementation of boundary condition critical for the microflow simulation. For the wall boundaries, we apply the combined bounce-back and specular-reflection (CBBSR) boundary condition. The slip boundary conditions are given in the following form:

(31) $$f_{i}=r_{b}f_{i}^{BB}+(1-r_{b})f_{i}^{SR}$$

where $f_{i}^{BB}(x_{w},t)$ and $f_{i}^{BR}(x_{w},t)$ are the known distribution functions of the node $x_{w}$ at the wall corresponding to the directions of specular reflection and bounce-back, respectively. $r_{b}$ is the reflection coefficient with a value between zero and one, $r_{b}$ and $1-r_{b}$ represent the proportion of bounce-back reflections and the specular-reflections proportion, respectively. In the past investigations, many researchers have done a lot of work on implementation of slip boundary condition [28,29,34,36,46,49,50,51]. All the studies have highlighted the crucial role played by boundary conditions. Different values of $r_{b}$ were taken to describe the impact factors of gas flow in microchannels like rarefaction effect, velocity slippage, surface diffusion and roughness and so on. Guo et al. [34,49] pointed out that $r_{b}$ was a function of the Knudsen number, and proposed a corrected combination coefficient including the contribution of the effects of adsorbed layers and Knudsen layers. Duan [50] pointed out that slip flow at the wall had a quadratic dependence on the Knudsen number. Then he employed the appropriate or effective second-order slip coefficients considering the Knudsen layers to capture the slip motion on the wall. There does not exist a general or exact value of $r_{b}$ to deal with the boundary conditions in the hydrodynamic fields, and the choice of $r_{b}$ is left with some ambiguity. In our work, the value for $r_{b}$ is selected to be 0.7 in the slip regime. Arkilic et al. [7] have revealed that the good agreement between the analytical solutions obtained from the Navier–Stokes equations and experimental data. Tang et al. [46] pointed out that the theoretical value agreed well with the experimental value [7] under the smooth surface boundary condition in the slip regime when the slip coefficient was 0.7.

## 4. Simulation Results and Discussion

### 4.1. Developing Velocity Profiles

**Figure 2:**
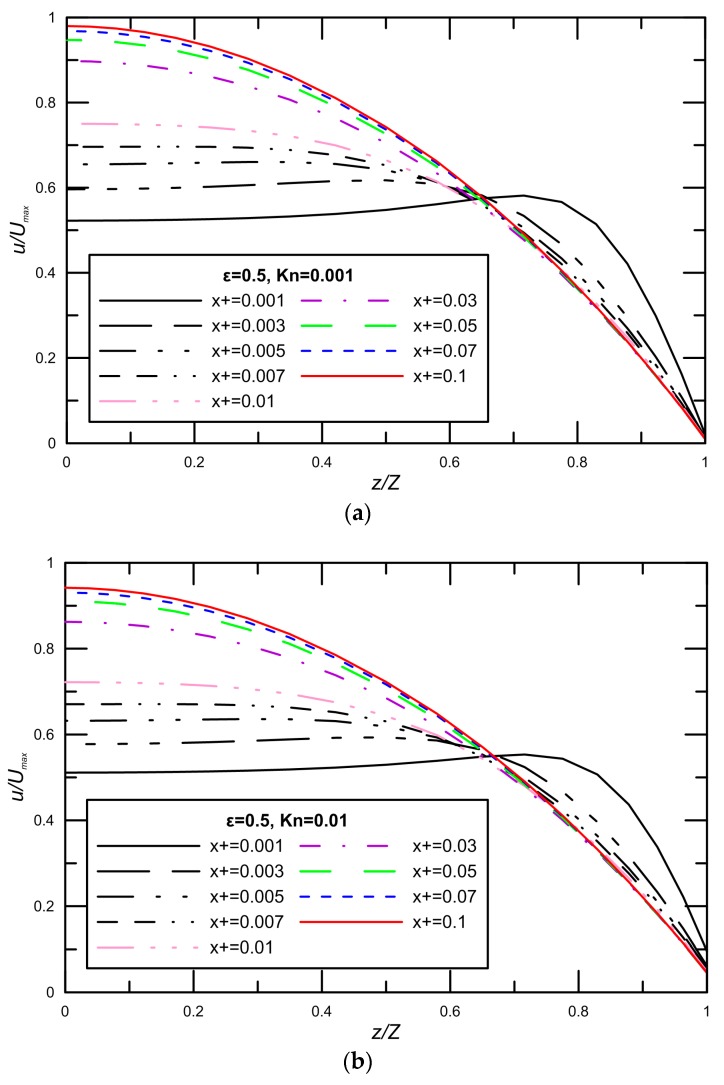

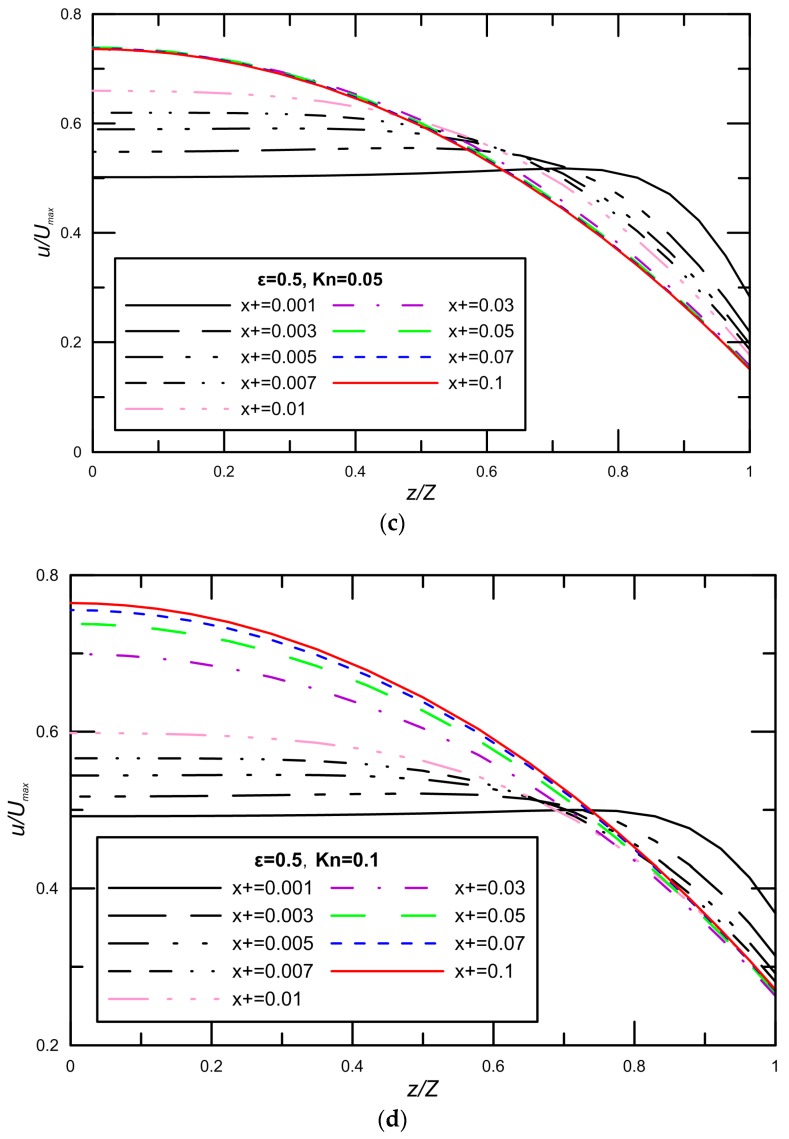
Developing velocity profiles in the streamwise direction of the microchannel in the slip regime.

The development of the three dimensional, laminar velocity profiles in the entrance region of a rectangular duct are investigated, the velocity profiles for aspect ratio ε=0.5 with varying Knudsen numbers are shown in Figure 2. The plots are normalized in the streamwise direction *x* with the microchannel width on the *z*-axis, and with the theoretical maximum centerline fully-developed axial velocity on the *x*-axis $u/u_{\max}$. This figure shows the three-dimensional structures of different velocity profiles in their deformation from a uniform inlet velocity profile to a parabola with the minimum velocity in the core region and maximum velocity at the wall. It is found that if the axial velocity profile at the inlet is uniform, it subsequently develops overshoots, and this is clearly observed in Figure 2. The phenomenon leading to the velocity overshoots may be explained as follows. Near the entrance where the fluid particles first meet the wall, viscous friction rapidly decelerates the flow to zero velocity at the wall. The high velocity gradient at the wall results in a high shear stress and the high pressure gradient needed to produce the high acceleration in the near wall region. Therefore, the flow near the centerline is not accelerated immediately, whereas the flow near the wall is forced to be stationary as soon as it enters the entrance region. The velocity overshoots are thus formed in order to satisfy the continuity equation. The effects of the Kn on the velocity profile at the developing region are also analyzed. For high Kn = 0.1 there are still slight overshoots at the axial locations close to the inlet. Additionally, note that the amount of slip velocity at a given Kn decreases as the flow approaches the fully-developed region due to the reduction in the velocity gradients at the wall. Furthermore, increasing Kn causes to decreasing maximum velocity at the middle of microchannel, but the velocity near the wall augments due to an increase in the slip velocity. At a given flow rate, the velocity profiles in a microchannel for high Kn are more uniform than low Kn.

### 4.2. The Effects of Aspect Ratio and Knudsen Numeber on the $f_{app}Re$

As demonstrated in the analytical section, the pressure-drop results are presented conveniently in terms of the grouping apparent friction factor and Reynolds number product $f_{app}Re$. Accurate evaluation of pressure-drop and analyzing the effect of rarefaction on pressure-drop are critically paramount for designing compact microchannel heat sinks and exchangers.

In this part, we use the calculated results from Equation (10) in Duan and Muzychka [45] as the reference to compare with present study. We also present a comparison between the finite volume method and LBM, as extracted from numerical data. To take into account the entrance effects on the overall friction factor in the microchannel, the dimensionless developing length $x^{+}=x/D_{h}Re$ is the proper parameter.

**Figure 3:**
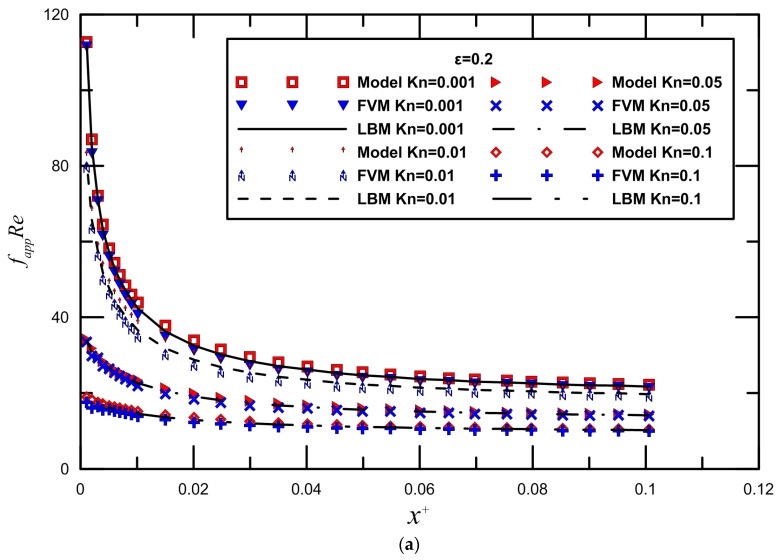

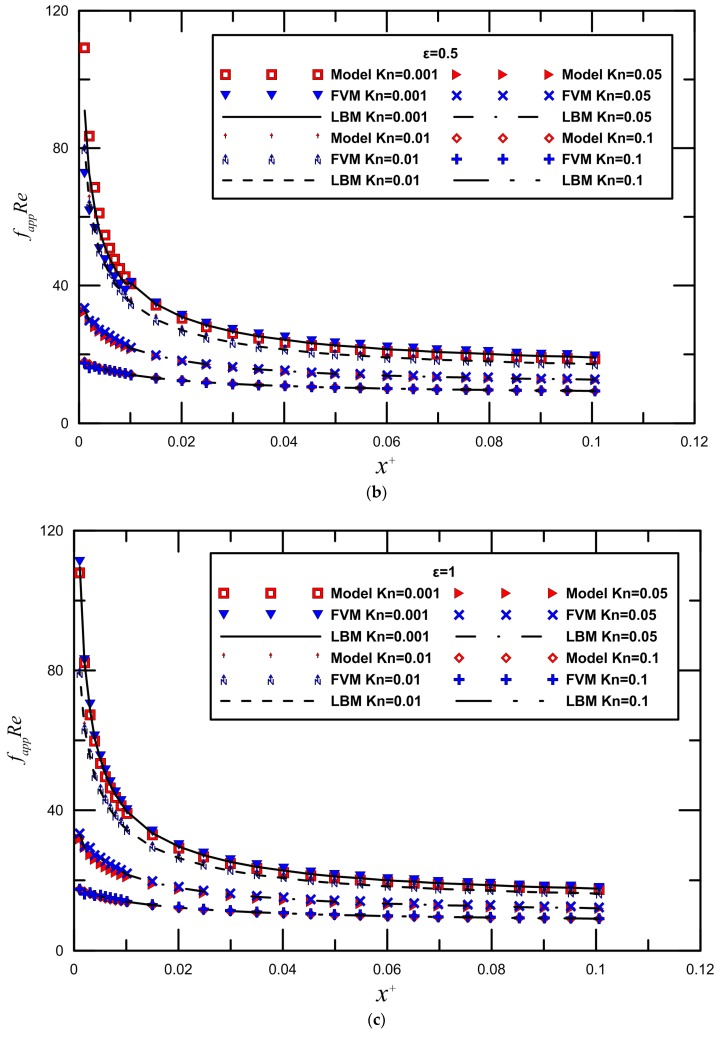
Comparisons between semi-theoretical model and FVM with three different aspect ratios.

Due to the relatively short lengths employed in microchannels, the influence of the entrance region cannot be neglected. The entrance region effects become more pronounced at smaller Knudsen numbers in part explaining the trend of decreasing $f_{app}Re$, as seen from Figure 3, in which the apparent friction factor and Reynolds number product $f_{app}Re$ losses along the streamwise direction with three different aspect ratios 0.2, 0.5, and 1 in the slip regime are investigated. We can see that with the increase of the axial dimensionless distance $x^{+}$, $f_{app}Re$ gradually reaches the full stage stability value. The rarefaction effect due to increasing of Kn can also be observed from these plots. The effect of rarefaction was assessed by varying the Knudsen number, which shows a decrease with an increase in Kn. For the same aspect ratio, the $f_{app}Re$ decreases with increasing Knudsen number. This trend is reasonable since the flow is more rarefied at higher Knudsen numbers. The reduction of the $f_{app}Re$ is stronger for rectangular microchannels with smaller channel aspect ratios. It can be seen that fully-developed flow is attained at different *x*^+^ values, with the low aspect ratio ducts reaching it earlier. As described earlier, initially, the change in Knudsen number (due to the density variation) plays a vital role in determining the friction coefficient behavior. In addition, compared with the traditional numerical methods for microflow simulation, such as the finite volume method, the computation effort of the lattice Boltzmann method may increase. An excellent agreement between the numerical and analytical results in the range 0.001 < Kn < 0.1 is clearly observed, such that the differences are found to be within 8.5%.

**Figure 4:**
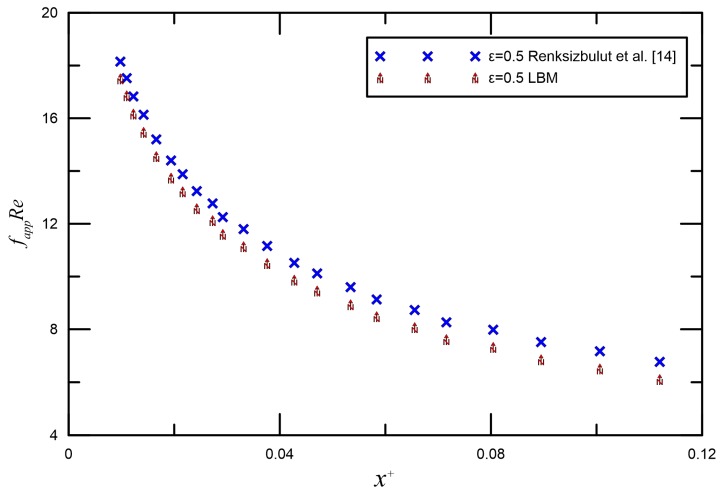
Comparison of $f_{app}Re$ for the numerical data by Renksizbulut et al. [14] for *ε* = 0.5 in the continnum flow regime.

**Figure 5:**
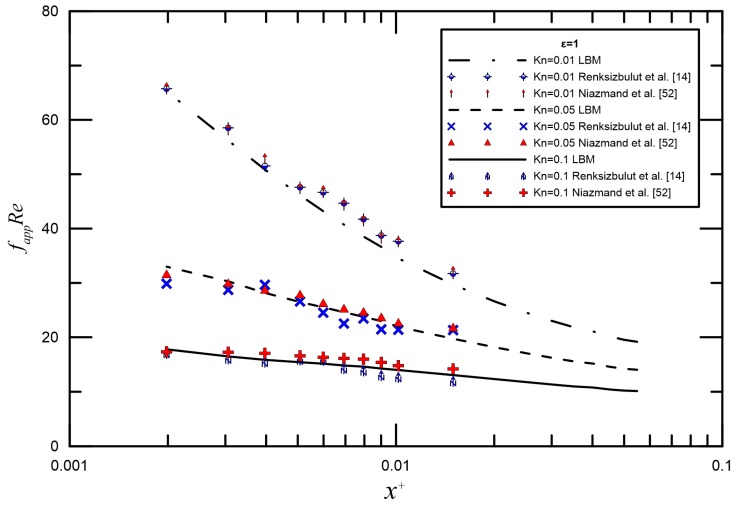
Comparison of $f_{app}Re$ for the numerical data by Renksizbulut et al. [14] and Niazmand et al. [52] for *ε* = 1 in the slip flow regime.

Slip flow in the entrance region of rectangular microchannels was investigated numerically by Renksizbulut et al. [14] and Niazmand et al. [52]. Figure 4 and Figure 5 demonstrate the comparison between LBM reports and the numerical data by Renksizbulut et al. [14] and Niazmand et al. [52] for different Knudsen numbers. Good quantitative agreement was observed between the simulations and previous numerical data from [14,52]. The maximum deviation is generally less than 10%.

### 4.3. Uncertainty Analysis

**Figure 6:**
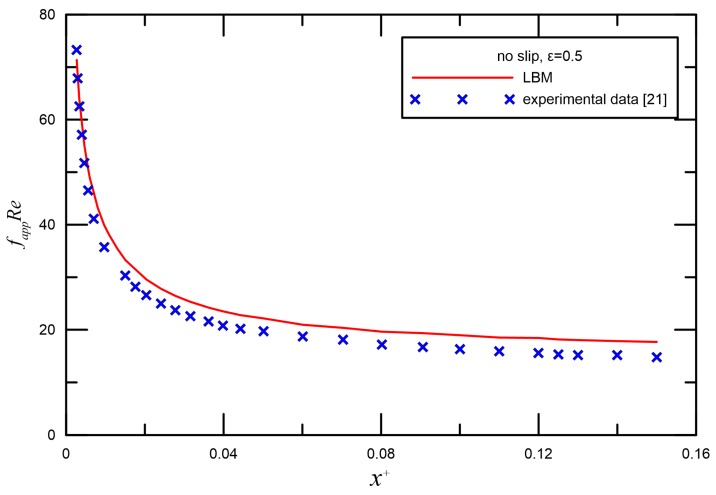
Comparison of $f_{app}Re$ at the aspect ratio of 0.5 (Kn→0).

Figure 6 shows that when a gas flows through a microchannel, the deviations of the apparent friction factor and Reynolds number product from the results obtained resorting to LBE method and experimental data [21] for continuum flow (Kn→0). The difference between LBM results and experimental data [21] is from 7.5% to 10%.

To account for this discrepancy, a careful analysis of the experimental uncertainty in this study is critical to the interpretation of experimental data and exploration of deviation from the LBE method. An uncertainty analysis is performed on relevant parameters using the root sum square method described by Moffat [53]. Assuming *U* is a function of the independent variables $x_{1}$, $x_{2}$, $x_{3}$, …, $x_{n}$, and $\Delta x_{1}$, $\Delta x_{2}$, $\Delta x_{3}$, …, $\Delta x_{n}$ are the uncertainties in these independent variables, the uncertainty of *U* can be estimated by Equation (32):

(32) $$\Delta U={\left[{\left(\frac{\partial U}{\partial x_{1}}\Delta x_{1}\right)}^{2}+{\left(\frac{\partial U}{\partial x_{2}}\Delta x_{2}\right)}^{2}+{\left(\frac{\partial U}{\partial x_{3}}\Delta x_{3}\right)}^{2}+\ldots +{\left(\frac{\partial U}{\partial x_{n}}\Delta x_{n}\right)}^{2}\right]}^{1/2}$$

Uncertainty analysis provides a reasonable method to evaluate the significance of the scatter on experiments. This is a powerful tool in locating the source of trouble in an experiment.

For incompressible flow through horizontal pipes or channels of constant cross-sectional area, the friction factor and Reynolds number product can be expressed as follows:

(33) $$fRe=\frac{\pi }{8}\frac{\Delta PD_{h}{}^{4}}{Q\mu L}$$

Equation (34) offered for the discrepancy of the fully developed laminar flow’s predictions and that obtained for an incompressible developing flow with the entrance section effects. The final expression for uncertainty in estimating $f_{app}Re$ is given by:

(34) $$f_{app}Re=\frac{\pi }{8}\frac{\Delta PD_{h}{}^{4}}{Q\mu L}+\frac{1}{\pi }\frac{\rho Q}{L\mu }\sum K_{L}(x^{+})$$

According to Equation (35), the experimental uncertainties in $f_{app}Re$ may be expressed as:

(35) $$u_{f_{app}Re}=\frac{\pi }{8}\frac{\Delta PD_{h}{}^{4}}{Q\mu L}{\left\{\begin{array}{l} {\left(\frac{u_{\Delta P}}{\Delta P}\right)}^{2}+{\left(4\frac{u_{D_{h}}}{D_{h}}\right)}^{2}+{\left(1-\frac{Re}{C^{*}}\frac{D_{h}}{L}\sum K_{L}(x^{+})\right)}^{2}{\left(\frac{u_{Q}}{Q}\right)}^{2}\\ +{\left(1+\frac{Re}{C^{*}}\frac{D_{h}}{L}\sum K_{L}(x^{+})\right)}^{2}\left[{\left(\frac{u_{\mu }}{\mu }\right)}^{2}+{\left(\frac{u_{L}}{L}\right)}^{2}\right]+\left(\frac{Re}{C^{*}}\right)2{\left(\frac{D_{h}}{L}\right)}^{2}u_{K_{L}}^{2} \end{array}\right\}}^{1/2}$$

where Q is the fluid volumetric flow rate and μ is the dynamic viscosity. *K_L_*(*x*^+^) represents the losses due to the hydrodynamic developing region. The product of the measured friction factor and Reynolds number can then be compared to the theoretical value for laminar flows. For flows in ducts of various cross-sections, a relationship of the form $fRe=C^{*}$ exists, where $C^{*}$ is a constant dependent only on the channel geometry.

It can be seen that the most dominant terms in the $f_{app}Re$ uncertainty are the measurements of the microchannel hydrodynamic diameter and the determination of developing region effects. The contradictions between experimental results and the LBE method are due to factors involving experimental error and the lack of accounting for the influence of entrance region. As a matter of fact, for fluid flow in microchannels, the effect of entrance region is relatively complex and difficult to involve while the dimensions of channels approach microlevel. In addition, the propagation of errors in the system can become troublesome.

### 4.4. Hydrodynamic Development Length

The flow-developing region is very important, particularly in microchannels, when dealing with fluid flow within micro-channels, as these ducts are usually very short. In most applications, the short length of the channels is not enabling the flow to reach the fully developed regime. Thus, the length of the hydrodynamic developing region $L^{+}=L/D_{h}Re$ is useful when designing a microfluidic device to account for the entrance region effects or choosing an appropriate location for the upstream boundary of an accurate numerical model. Assuming the entering flow is uniform at the channel inlet, the hydrodynamic entrance length $L^{+}$ is defined as the axial location where maximum velocity attains 99% of the corresponding fully developed value.

Several hydrodynamic entrance length studies have been explored both experimentally and numerically. Regarding numerical studies for rectangular ducts and parallel plate channels, results found by groups, such as Han [54], Fleming and Sparrow [55], Atkinson et al. [56], Wiginton and Dalton [57], and Chen [58], are considered to relatively standardly and accurately analyze the entrance length. Many entrance length studies for circular ducts have also been investigated by some groups, such as Sparrow et al. [59], Goldstein and Kreid [60], Beavers et al. [61], and Muchnik et al. [62].

For low Reynolds numbers one can consider entrance length correlations given by Atkinson et al. [56], Chen [58], Durst et al. [63] and Duan and Muzychka [44], for parallel plates. The parallel plate correlation of Atkinson et al. [56] was given by:

(36) $$\frac{L}{D_{h}}=0.625+0.044Re$$

Chen [49] proposed Equation (37) basing on the solutions of Atkinson et al. [56]:

(37) $$\frac{L}{D_{h}}=\frac{0.63}{0.035Re+1}+0.044Re$$

Durst et al. [63] employed numerical, experimental, and analytical methods to study the development lengths of channel flows. They proposed a linear relationship between the entrance length and Reynolds number, as shown in Equation (38). However, this correlation did not correctly describe the development length in the region 1 < *Re* < 100:

(38) $$\frac{L}{D_{h}}={\left[{\left(0.631\right)}^{1.6}+{\left(0.0442Re\right)}^{1.6}\right]}^{1/1.6}$$

Duan and Muzychka [44] applied a curve fit in the form of the correlation of Chen [58] at the low *Re*, which considered the effects of slip flow:

(39) $$\frac{L}{D_{h}}=\frac{0.315}{0.0175Re+1}+0.0112Re\left[1+6.7\frac{2-\sigma }{\sigma }Kn-37{\left(\frac{2-\sigma }{\sigma }Kn\right)}^{2}\right]$$

These correlations were established in common through a combination of the creeping flow and boundary-layer type solutions. Looking at these above correlations, when *Re* is high, an approximately linear curve is depicted. However, at very low *Re*, $L/D_{h}$ goes towards a constant value and is independent of *Re*.

In a rectangular microchannel, four boundary layers are created on each wall beginning at the channel inlet. It is similar to the boundary layer on a flat plate. They finally merge at some point downstream. The numerical correlations given by Han [54] and Wiginton and Dalton [57] are linear correlations, where the entrance length is proportional to the Reynolds number by some constant, depending on the cross-sectional aspect ratio. Renksizbulut and Niazmand [64] studied, numerically, the laminar flow and heat transfer in the entrance region of trapezoidal and rectangular channels. The correlation for the entrance length was estimated to be about 15% accurate for 10 < *Re* < 1000; however, a limited range of rectangular channel aspect ratios 0.5<ε<2 was investigated. Ahmad and Hassan [65] carried out an experimental investigation to study the hydrodynamics in the entrance region of microchannels using microparticle image velocimetry (micro-PIV). The experiments were performed in the laminar flow region with a *Re* range of 0.5–200. Galvis et al. [66] investigated numerically the effects of aspect ratio and hydraulic diameter on the hydrodynamic entrance length in rectangular microchannels. The numerical simulations were performed to evaluate the effects of Reynolds number (50 < *Re* < 200), hydraulic diameter ($0.1\;\mathrm{mm}<D_{h}<0.5\;\mathrm{mm}$), and channel aspect ratio (0.2<ε<1) on the entrance length in rectangular microchannels. They reported that the channel aspect ratio has an insignificant effect on the dimensionless entrance length for *Re* > 50. New correlations were proposed to predict the entrance length for rectangular microchannels with 0.2<ε<1 and for *Re* < 50.

For square microchannels, the correlation of Han [54] was given by:

(40) $$\frac{L}{D_{h}}=0.0752Re$$

The correlation of Wiginton and Dalton [57], for an aspect ratio of 1, was given by:

(41) $$\frac{L}{D_{h}}=0.09Re$$

Renksizbulut and Niazmand [64] suggested a correlation for a limited range of aspect ratios 0.5 < *ε* < 2 and Reynolds number 10 < *Re* < 1000:

(42) $$\frac{L}{D_{h}}=\left[0.085Re+\frac{0.8}{Re^{0.3}}\right]\left[{\left(1+\varepsilon \right)}^{-0.24}\right]$$

Ahmad and Hassan [65] proposed a general empirical correlation for macro and microchannels to estimate the dimensionless entry length, as given by Equation (43):

(43) $$\frac{L}{D_{h}}=\frac{0.74}{0.09Re+1}+0.0889Re$$

Galvis et al. [66] proposed a new correlation to predict the entrance length for rectangular microchannels with 0.2<ε<1 and for *Re* < 50:

(44) $$\frac{L}{D_{h}}=\frac{0.60}{0.14Re+1}+0.0752Re$$

**Figure 7:**
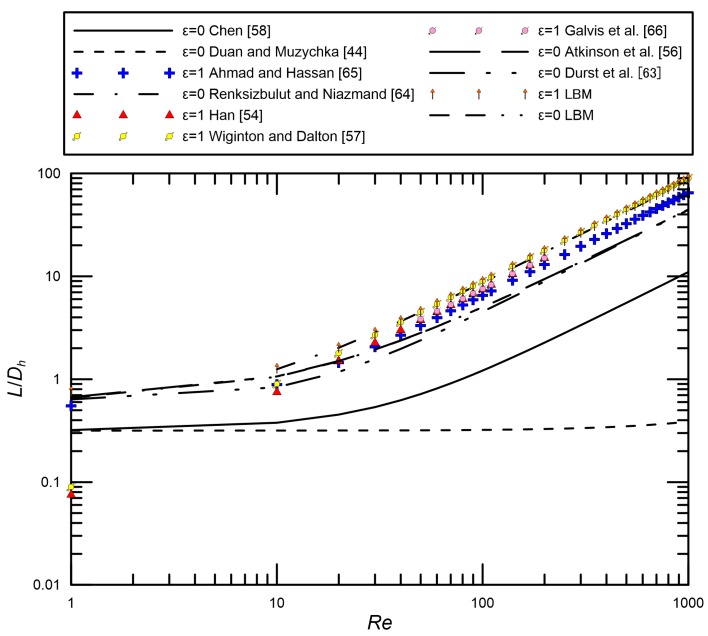
Comparison of current data and correlations for entrance length from the literature.

A comparison of entrance length data obtained from LBM and several correlations in previous studies for square microchannels and parallel plate channels is presented in Figure 7. It is evident that the universal entrance length correlation does not exist for the rectangular channels of various aspect ratios and different Knudsen numbers over the entire range of Reynolds numbers common to practical devices. Therefore, our present work is focused on a new developing correlation that can be used to estimate the entrance length in rectangular microchannels that contains the effects of aspect ratios and Knudsen numbers for a wide range of Reynolds numbers 1 < *Re* < 1000, including creeping flows.

**Figure 8:**
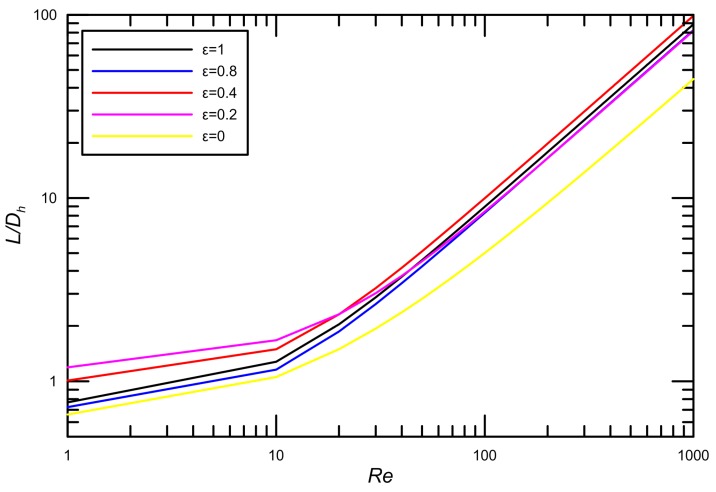
Variation of $L/D_{h}$ with *Re* for different aspect ratios.

First, the effects of Reynolds numbers and aspect ratios on nondimensional hydrodynamic entry length are analyzed in Figure 8. The results show that the hydrodynamic entry length $L/D_{h}$ increases nonlinearly with the increase of Reynolds number, at low *Re* number, the developing length is very small and the flow becomes fully developed over a significant part of the channel length.

**Figure 9:**
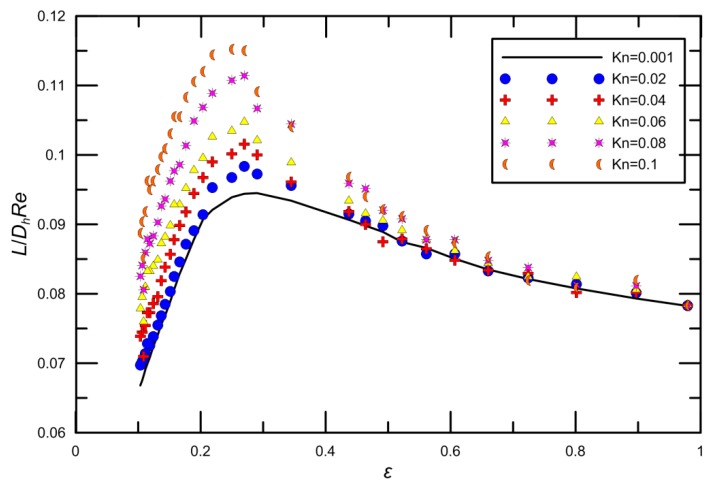
Variation of entrance length with channel aspect ratio *ε* at different *Kn* values.

It can be seen from Figure 9 that $L^{+}=L/D_{h}Re$ increases initially and decreases later with the increase of the aspect ratio ε. The rarefaction effects on the entrance length are also contained in Figure 9. For the range of *Re* considered in the present study, entrance lengths display a highly nonlinear dependence on the channel aspect ratios. Namely, a certain aspect ratio exists that contributed to the maximum value of $L^{+}$. The certain ε can be different with various Knudsen numbers. When ε is small, the hydrodynamic diameter is also small. This is because the variation in the channel geometry due to the variation of aspect ratio changes the surface effect in channel. The Knudsen number has an increasing effect on the entrance length for all aspect ratios.

All figures serve to illustrate the role of the Reynolds number, aspect ratio, and Knudsen number on the hydrodynamic entrance length. Based on the present results, we present a relationship for the entrance length in rectangular microchannels under the slip condition. The form of the function we used is as follows:

(45) $$L/D_{h}=\left[\frac{a}{bRe+1}+cRes(Kn)\right]f(\varepsilon )$$

where a, b, c are constant, s(Kn) and f(ε) are used to relatively describe the relationship between $L/D_{h}$ and Knudsen number Kn, $L/D_{h}$ and aspect ratio ε. Here, s(Kn) and f(ε) are expressed as polynomial forms.

Through curve fitting, the values of a, b, c and the expression of f(ε) are obtained.

(46) $$\begin{array}{ll} L/D_{h}= & \left[\frac{0.60}{0.072Re+1}+0.089Re(0.01853+0.017293Kn-0.057125Kn^{2})\right]\\ & \left(0.0632+0.1095\varepsilon -0.1051\varepsilon^{2}\right) \end{array}$$

Equation (46) indicates that the entrance length has a rational relationship with the Reynolds number when the Reynolds number is small. The entrance length does not vanish as *Re* approaches zero. The entrance length increases as the aspect ratio increases until it reaches a maximum near an aspect ratio of 0.3 and then it decreases as the aspect ratio increases further. The entrance lengths at the higher Reynolds numbers could become quite significant for comparatively short microchannels. Furthermore, microscale effects could possibly lengthen the entrance region.

Table 1 shows the comparison of $L/D_{h}$ at different Knudsen numbers between the present results and the values reported by Hettiarachchi et al. [67]. It is found that the correlation agrees with the available data well, which shows that this correlation can predict hydrodynamic entry length well. It confirms that the correlation we obtained here is reliable.

## 5. Conclusions

In this paper, we have performed an investigation in slip flow through a rectangular microchannel using an improved incompressible LBGK D3Q15 model. The combined effects of aspect ratios and rarefaction on friction distribution characteristics have been investigated by employing the model. Our numerical results show that the entrance friction factor and Reynolds number product is of finite value and that the friction factor and Reynolds number product reduces with the decrease in Knudsen numbers and the increase in the channel aspect ratios. Various comparisons are presented among the available literature, including the numerical, experimental, and the semi-theoretical analytical data, which are in excellent agreements. Note that the solutions of the linearized Boltzmann equation are remarkably close to analytical results by solving the Navier–Stokes equation with slip boundary conditions in the slip regime. This means that the lattice Boltzmann method with a scheme of combining the bounce-back reflection with specular reflection applying to the boundary condition treatment can be capable of predicting the slip flow at the entrance region well.

In addition, numerical simulations have been used to study the developing flows in rectangular microchannels, focusing on the entrance length and its dependence on the Reynolds number and channel aspect ratio. Novelty correlation for the entrance length was proposed for rectangular channels with 0.1<ε<1 and 1 < *Re* < 1000 under slip condition. The entrance length generally increases with the increase of Kn while the channels with aspect ratio near 0.3 are shown to have the largest entrance length.

The results presented here are remarkable because they uncover the effects of rarefaction and aspect ratios on flow behavior at the entrance region in the slip regime which were not systematically reported earlier. These detailed results can also be used for benchmarking future microdevice designing. Furthermore, the present study serves as a baseline case for understanding transition flow in complex microchannels.

## Figures and Tables

**Table 1:** Comparison of $L/D_{h}Re$ at different Knudsen numbers between the present results and Hettiarachchi et al. [67] for ε=0.2,0.5,1.

*Kn*	*L*/*D_h_ Re*								
	*ε* = 0.2			*ε* = 0.5			*ε* = 1		
	Present Results	Data [67]	Error (%)	Present Results	Data [67]	Error (%)	Present Results	Data [67]	Error (%)
0.001	0.07825	0.07782	0.55	0.08892	0.08858	0.38	0.09073	0.09028	0.50
0.02	0.07835	0.07791	0.56	0.08980	0.08942	0.42	0.09530	0.09483	0.49
0.04	0.07844	0.07801	0.55	0.09027	0.08974	0.59	0.09899	0.09847	0.53
0.06	0.07874	0.07836	0.48	0.09054	0.09025	0.32	0.10258	0.10226	0.31
0.08	0.07925	0.07885	0.50	0.09203	0.09163	0.43	0.10889	0.10841	0.44
0.1	0.08026	0.07985	0.51	0.09212	0.09164	0.52	0.11442	0.11407	0.31

## Nomenclature

A	Flow area (m^2^)
a	Minor semi-axis of rectangle (m)
b	Major semi-axis of rectangle (m)
c	Particle streaming speed
$c_{i}$	Discrete velocity
$c_{s}$	Speed of sound (m/s)
$C^{*}$	Constant =fRe
$D_{h}$	Hydraulic diameter = 4A/P (m)
f	Fanning friction factor = $\tau_{\omega }/(1/2\rho u_{m}^{2})$
$f_{app}$	Apparent friction factor accounting for developing flows dimensionless developing flows, dimensionless
$f_{i}(x,t)$	Particle distribution function
$f_{i}^{eq}$	Local-equilibrium distribution function
Kn	Knudsen number = $\lambda /D_{h}$
$Kn^{*}$	Modified Knudsen number (for liquids) = $\lambda_{s}/D_{h}$
K(∞)	Hagenbach’s factor
L	Hydrodynamic entrance length (m)
$L^{+}$	Dimensionless hydrodynamic entrance length = $L/D_{h}Re$
N	Grid number in the characteristic length
n	Normal coordinate
P	Perimeter (m)
p	Pressure (N/m^2^)
$Po_{app}$	Apparent Poiseuille number
Q	Fluid volumetric flow rate (kg/s)
R	Specific gas constant (m^2^/(s^2^∙K))
$r_{b}$	Reflection coefficient
*Re*	Reynolds number = $\rho u_{m}D_{h}/\mu$
$T_{0}$	Reference gas temperature (K)
U	A function of the independent variables
$u_{f_{app}Re}$	Uncertainty in estimating $f_{app}Re$
$u_{m}$	Average velocity (m/s)
$u_{\max}$	Maximum velocity (m/s)
u,v,w	Velocity components (m/s)
$x^{+}$	Dimensionless channel length = $x/Re/D_{h}$
x	Coordinate in flow direction (m)
$x_{i}$	Independent variable
y,z	Cartesian coordinates (m)
Greek Symbols	
$\alpha_{i}$	Eigenvalues
ε	Aspect ratio = *a*/*b*
λ	Molecular mean free path (m)
$\lambda_{s}$	Slip length (m)
ν	Kinematic viscosity (m^2^/s)
μ	Dynamic viscosity (Ns/m^2^)
ρ	Density (kg/m^3^)
σ	Tangential momentum accommodation coefficient
τ	Dimensionless collision relaxation time
Subscripts	
app	Apparent
BB	Bounce-back
eq	Equilibrium
m	Mean
max	Maximum
SR	Specular-reflection
w	Weighting coefficient
wall	Wall surface
